# Microbiome-mediated resilience and cross-generational consequences in male *Drosophila* exposed to combined environmental stressors

**DOI:** 10.1098/rspb.2026.1300

**Published:** 2026-10-14

**Authors:** Komal Maggu, Abhishek Meena, Alessio N. De Nardo, Benjamin Eggs, Sonja H. Sbilordo, Rawaa Al Toma Sho, Stefan Lüpold

**Affiliations:** Department of Evolutionary Biology and Environmental Studies, University of Zurich, Zürich, 8057, Switzerland

**Keywords:** microbiome, thermal fertility, pesticide exposure, inter-generational effects, stress resilence, sperm competition

## Abstract

Climate change exposes organisms to multiple, interacting stressors, yet their joint effects on reproduction and inheritance remain poorly understood. This study investigated the interactive effects of acute heat stress and sublethal imidacloprid exposure on the reproductive success of male *Drosophila melanogaster* and the subsequent fitness of their offspring. We manipulated the gut microbiome of male flies (germ-free or colonized with one or five bacterial species) and subjected them to individual or combined stress. We show that combined stressors act synergistically to reduce male fitness traits, an effect that is partially buffered by higher microbiome diversity. Paternal stress exposure further reduced offspring fertility, demonstrating cross-generational fitness costs largely independent of microbial transmission. Our results underscore the gut microbiome as an important, but stress-sensitive, contributor to host resilience, and demonstrate how multi-stressor interactions can amplify reproductive failure, with broad implications for population persistence and biodiversity loss in a warming, chemically altered world.

## 1. Introduction

The increasing prevalence of human-induced stressors poses a significant challenge to global biodiversity [1–5], with climate change and pesticides being key drivers of biodiversity loss and their interaction presenting unique ecological risks [6–8]. Thermal stress impairs cellular function [9], disrupts reproductive physiology [10,11] and can push ectotherms close to their thermal limits [12], while pesticide exposure impairs development, behaviour and fertility in both target and non-target species [13,14]. Although the independent effects of these stressors have been studied extensively [14,15], natural systems rarely experience them in isolation. When combined, stressors often interact in non-additive ways, producing outcomes that are synergistic or antagonistic rather than predictable from single-stressor responses [16,17]. Such interactions make it difficult to forecast the performance of individuals and the persistence of populations under global change, highlighting the importance of studies that explicitly test stressor combinations.

Another critical knowledge gap concerns stress effects on male reproduction. Male sterility frequently occurs at temperatures well below those causing mortality [18–20], such that fertility performance curves are narrower than viability curves [21] and reproductive limits are more reliable predictors of climate vulnerability than classical tolerance thresholds [18,20,22]. This heightened sensitivity is not restricted to temperature: both thermal and chemical stressors can disrupt spermatogenesis, sperm DNA integrity and ejaculate quality [15,23,24], traits central to fertilization success, particularly under sperm competition [25–28]. Because sperm production and function are highly condition-dependent, male reproductive failure may precede detectable effects on survival and accelerate population decline under environmental stress [22,29]. Moreover, heat stress and neonicotinoid exposure may converge mechanistically on neuromuscular function: elevated temperatures disrupt neuronal performance and membrane stability [30], while imidacloprid acts as an agonist of insect nicotinic acetylcholine receptors, thereby impairing cholinergic signalling [23]. Such overlap suggests that thermal and chemical stressors may interact through shared physiological pathways and potentially amplify their effects on behaviour and reproductive performance. Because reproductive traits also shape the strength and direction of selection [24], understanding how multiple stressors disrupt male reproduction is essential for predicting adaptive responses and population persistence under global change.

It is becoming increasingly evident that host responses to environmental stress can be mediated by associated microbial communities, including facultative and environmentally acquired gut microbiota [25–28]. In *Drosophila*, where the microbiome is dominated by extracellular *Acetobacter* and *Lactobacillus* species, gut bacteria have been shown to influence thermal performance, heat tolerance and recovery from temperature stress [27,31]. Thermal exposure itself can strongly reshape microbial abundance and community structure, highlighting a dynamic, bidirectional relationship in which gut microbiota both contribute to host thermal resilience and constitute a stress-sensitive component of host physiology [32–35]. Commensal microbes can also increase insect resistance to insecticides through direct pesticide metabolism or by inducing the expression of detoxification genes in the host [36–39]. Despite progress in studying individual stressors, how microbiomes modulate *combined* stressor effects remains largely unknown. Understanding these dynamics is critical, as microbiomes may provide resilience but may also constitute an axis of vulnerability if disrupted.

Additionally, sperm damage can also extend the environmental stress effects across generations through epigenetic modifications or sperm-borne small RNAs [40,41], affecting population stability and evolution [42,43]. While parental exposure to combined thermal and chemical stress has been shown to reduce offspring performance in some species [44,45], other studies find no clear effects [17,46] or even reduced offspring vulnerability [47], indicating interspecific variation due to stressor intensity (including the thermal sensitivity of pesticides themselves), species-specific acclimation or compensatory maternal provisioning. Understanding these cross-generational impacts is crucial for predicting long-term ecological consequences and evolutionary adaptation potential.

To address these gaps, we used *Drosophila melanogaster* as a tractable model to examine how acute thermal stress and pesticide exposure interact with host–microbiome associations to shape male reproductive fitness and its inheritance. Although *D. melanogaster* may not encounter imidacloprid at ecologically relevant concentrations in the wild, it is a well established model for investigating neonicotinoid effects on insect physiology [48], and neonicotinoids target conserved nicotinic acetylcholine receptors whose temperature-dependent modulation of toxicity has been documented across diverse insect taxa [49–51]. Our use of *D. melanogaster,* therefore, allowed us to examine generalizable mechanisms of pesticide–temperature interactions and their modulation by gut microbiome composition under tightly controlled conditions, with relevance to pest and non-target insects that experience both stressors in agricultural landscapes.

We isolated microbial contributions by employing a gnotobiotic framework that compared axenic males with those colonized by *Acetobacter tropicalis*, *Lactobacillus brevis* or a five-species consortium of *Acetobacter* and *Lactobacillus* spp. representative of natural *Drosophila* microbiotas [52–56]. These taxa are commonly detected in laboratory and wild populations and are known to influence host metabolism, stress physiology and reproductive performance [53], including sperm competitiveness [56], which motivated their use in the present study. Mono-associations permitted assessing taxon-specific effects, while the five-species consortium approximated a simplified natural community including both acetic acid and lactic acid bacteria [56]. This design allowed us to test three predictions: (i) multiple stressors act synergistically to reduce male reproductive fitness; (ii) commensal microbes buffer these effects in a species-specific manner, with enhanced protection emerging from multi-species communities through functional complementarity and redundancy [53,54]; and (iii) paternal exposure to combined stress reduces offspring fitness, with cross-generational effects attenuated in males harbouring more diverse microbiomes.

The rationale for predictions (ii) and (iii) draws on ecological theory and prior microbiome research. Different bacterial taxa may contribute distinct, partially overlapping functions, including nutrient provisioning, detoxification, immune priming and modulation of stress-response pathways, such that community-level diversity stabilizes host performance when individual strains are impaired by environmental stress, the so-called insurance hypothesis [57]. In *Drosophila*, multi-species gut communities have been shown to provide more robust and context-dependent benefits than mono-associations, particularly under variable environmental conditions [53,54]. For cross-generational effects specifically, we predicted that microbiome-mediated buffering of stress-induced damage to paternal physiology and gametes, including oxidative stress, metabolic disruption or immune dysregulation, would attenuate epigenetic or sperm-borne signals transmitted to offspring. We acknowledge that empirical evidence for paternal microbiome effects on offspring fitness in insects remains limited, and treat prediction (iii) as exploratory but biologically motivated.

## 2. Materials and methods

Detailed methods are available in the supplementary material, including an illustrated workflow in figure S1. The following is a concise description of the general experimental procedures.

### (a) Experimental population

We used an outbred *D. melanogaster* (LH_m_ wild-type) population maintained at 25°C, 60% humidity and a 12 h : 12 h light : dark cycle. To generate axenic (germ-free) focal flies, eggs collected within 6 h of oviposition were dechorionated using bleach and ethanol washes, then transferred to sterile glass vials containing autoclaved standard fly food supplemented with ampicillin, kanamycin, erythromycin and tetracycline. Axenic status of flies was confirmed by plating homogenates on Luria–Bertani (LB) and De Man–Rogosa–Sharpe (MRS) agar and by polymerase chain reaction (PCR) for bacterial DNA using universal 16S rRNA primers. The LH_m_ population is naturally infected with the endosymbiont *Wolbachia*. To verify infection status following the axenization procedure, we screened flies for the *Wolbachia* surface protein (*wsp*) gene by PCR and detected persistent infection despite antibiotic treatment.

### (b) Bacterial cultures and gnotobiotic associations

Axenic males were associated with defined gut microbiota isolated from wild-type flies [58]: mono-associations of *Acetobacter tropicalis* (*At*) or *Lactobacillus brevis* (*Lb*), or a five-species community (*A. tropicalis*, *Acetobacter orientalis*,* Acetobacter pasteurianus*,* Lactobacillus plantarum* and *L. brevis*). Bacterial strains were cultured overnight at 30°C in appropriate media (MRS + mannitol and MRS broth), centrifuged and resuspended in sterile phosphate-buffered saline (PBS) to a concentration of 10^8^ cells ml^–1^ [59,60].

Sterile food vials were inoculated with 50 µl of bacterial suspension (approx. 5 × 10^6^ CFU, with equal representation in the five-species consortium) [53,54]. Ten 1-day-old axenic males were added to each vial and transferred to fresh inoculated vials every 48 h for 4 days to ensure stable colonization. Successful colonization was confirmed by plating homogenates and checking for characteristic colony morphologies.

### (c) Stress treatments

After 4 days of colonization, males were transferred to sterile vials and subjected to a fully factorial design of pesticide and heat stress. Half of the males in each microbial group were exposed for 18 h to food containing 1 ppm imidacloprid, a concentration low enough to avoid immediate lethality while inducing sublethal effects on behaviour, reproduction and physiology [61–63]. Subsequently, half of the males from each microbiome and pesticide treatment were exposed to acute heat stress at 36°C for 4 h in vials containing 1% agar to prevent dehydration, while the remaining males were kept at 24.5°C as thermal controls. All males were then allowed to recover overnight in standard food vials under standard conditions (see ‘Experimental population’ (§2a)).

### (d) Hatching success and fertility

The day after stress treatments, each experimental male was mated with a randomly assigned virgin axenic female. Holding female microbiome status constant in this way prevented maternal microbial transmission and ensured that differences in reproductive output across treatment groups could be attributed to male condition rather than variation in female physiology. Following mating, females laid eggs for 24 h, and 30 h later, oviposition vials were frozen. Hatching success (proportion of hatched eggs) and male-mediated fecundity (total number of larvae) were then quantified. This assay was performed in two blocks with 25 males per treatment combination (i.e. a total of 800 males). However, the final analysed dataset comprised 650 observations after exclusions owing to unsuccessful mating trials, lack of oviposition or occasional handling losses.

### (e) Sperm competitiveness

Sperm competitiveness was assessed using a similar experimental design. Axenic, untreated females were each first mated with an axenic first male that ubiquitously expressed green fluorescent protein (GFP) [64,65]. Two days later, these females were allowed to individually remate with experimental males during an 8 h observation period. Remated females were then isolated in fresh vials with autoclaved food for two days of oviposition, while unmated pairs were given a second 8 h remating opportunity the following day before discarding those that still had not remated. Oviposition vials were incubated until adult emergence to determine paternity shares based on the GFP expression of first-male offspring under a fluorescence stereoscope. This assay included 30 remating trials per treatment combination in each of two blocks (i.e. total 960 trials), with 20.69 ± 2.15 trials per treatment combination (total *N* = 331), providing complete data for analysis.

### (f) Bacterial abundance in males

To assess the impact of stress on bacterial load, colony-forming units (CFUs) were compared in 10 random males per treatment combination. Surface-sterilized males were homogenized, serially diluted and plated on MRS medium. For groups with both *Acetobacter* and *Lactobacillus*, MRS agar was supplemented with ampicillin or incubated in a CO_2_ atmosphere for selective counting. Colony morphologies were used to identify bacterial species, and total species-specific CFUs per fly were calculated [59].

### (g) Cross-generational effects

To assess cross-generational effects of paternal stress, hatching success and fecundity were quantified in F_1_ offspring. From each block, up to 20 freshly hatched larvae from each of 10 randomly selected males per paternal treatment combination were transferred to vials containing sterile food and reared under standard conditions. Two to three days after eclosion, three virgin F_1_ males and females from each lineage were individually mated with axenically reared standard flies of the opposite sex. Hatching success and fecundity were then measured in these F_1_ offspring as for their fathers (above). This experiment involved two independent blocks of 480 offspring per sex from 160 F_0_ males (i.e. 960 mating trials per offspring sex in total, of which 725 and 740 trials generated complete analysable data for sons and daughters, respectively).

### (h) Statistical analyses

Data were analysed in R version 4.4.3 [66], using generalized linear mixed-effects models (GLMMs) with a Poisson (fecundity) or a binomial error distribution (hatching success, paternity shares), or using a linear mixed-effects model on the log scale in the case of bacterial abundance (CFUs). Models included microbiome composition, thermal and pesticide treatments (and their interactions) as fixed effects, while experimental block and, in the filial generation, sire were the random effects.

Microbiome community composition in the five-species consortium was analysed using Hellinger-transformed abundances and Bray**–**Curtis dissimilarities, PERMANOVA (*adonis2*) and environmental fitting (*envfit*), with non-metric multi-dimensional scaling (NMDS) (*metaMDS*) for visualization (all in the *vegan* package [67]). The goal of these analyses was not to infer ecological diversity or community complexity *per se* but to assess treatment-induced shifts in relative composition. Given the limited number of taxa, we focused on biologically interpretable abundance contrasts, particularly between acetic acid and lactic acid bacteria, which differ in metabolic function and stress sensitivity and are known to respond differently to temperature in *Drosophila* [33,34,68,69]. Additionally, relative abundances were compared at the genus level using a binomial GLMM accounting for block as a random effect and for overdispersion via observation-level random effect.

## 3. Results

### (a) Hatching success

To assess the combined effects of heat stress, pesticide exposure and microbiome composition on male reproductive function, we first investigated fertilization success, estimated as the proportion of eggs hatched out of all eggs laid by females. A binomial generalized linear model (GLM) revealed a significant three-way interaction between microbiome, temperature and pesticide (*N* = 650, *χ*² = 14.97, d.f. = 3, *p* = 0.002; figure 1A; supplementary material, table S1), highlighting that the combined effects of these stressors depended on the microbial composition of the focal males. Overall, males associated with all five bacterial strains exhibited the highest hatching success, followed by *At*- and *Lb*-associated males, with axenic males being most severely affected. These general patterns were also confirmed by the significant two-way interactions of temperature × microbiome (*χ*² = 21.89, d.f. = 3, *p* < 0.0001) and microbiome × pesticide (*χ*² = 18.15, *p* < 0.0001), as well as by all main effects (all *χ*² ≥ 67.43, *p* < 0.001; supplementary material, table S1).

**Figure 1.**
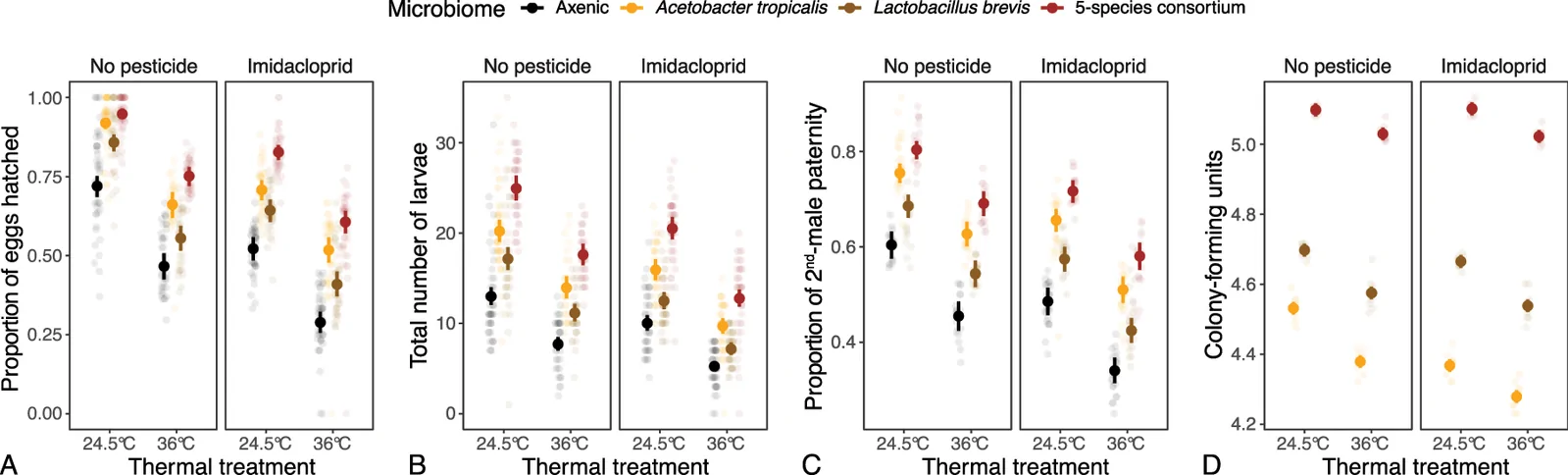
Effects of varying thermal and pesticide conditions and microbiome types on (A) hatching success, (B) male-mediated female fecundity, and (C) competitive fertilization success. (D) Bacterial abundance/colony-forming units of different microbial treatments (log_10_) in response to varying thermal and pesticide conditions. Semi-transparent points represent the raw data, and solid points with error bars the model-predicted means and 95% confidence intervals.

### (b) Male-mediated fecundity

Next, we investigated male-mediated fecundity, quantified as the total number of larvae produced per female. In contrast to hatching success, the three-way interaction was not significant (*χ*² = 0.69, d.f. = 3, *p* = 0.875) for this metric and was thus removed. The simplified model revealed significant two-way interactions between microbiome and temperature (*N* = 650, *χ*² = 8.06, d.f. = 3, *p* = 0.045) and between temperature and pesticide (*χ*² = 7.84, d.f. = 1, *p* = 0.005; figure 1B; supplementary material, table S2). All main effects were again significant (all *χ*² ≥ 23.94, *p* < 0.0001).

### (c) Sperm competitiveness

Given reports of adverse effects of raised temperatures and pesticide exposure on sperm quality and function [70–75], we evaluated the combined effects of our treatments on the proportion of progeny sired by focal males relative to their standard competitors (i.e. *P*_2_). Although none of the interactions was statistically significant and thus removed from the model (*N* = 331, all *χ*² ≤ 5.25, d.f. = 3, *p* ≥ 0.15), all experimental treatments exhibited highly significant main effects on *P*_2_ (all *χ*² ≥ 101.94, *p *≤ 0.0001; figure 1C; supplementary material, table S3).

### (d) Bacterial abundance in males in response to heat stress and pesticide exposure

We also analysed the effect of heat stress, pesticide exposure and their interactions on the growth and viability of commensal bacteria inside their host under the different stress treatments by quantifying their CFUs in homogenates of focal males immediately after mating. For total CFU counts, we found a significant three-way interaction (*N* = 120, *χ*² = 10.13, *p* = 0.006; figure 1D; supplementary material, table S4), suggesting that the bacterial strains differed in their accumulation patterns in adult flies depending on the combined effects of stress treatments. Additionally, we documented significant two-way interactions and main effects of all three factors (all *χ*² ≥ 12.10, *p* ≤ 0.001)

A PERMANOVA controlling for block effects (*F*_1,35_ = 0.20, *p* = 0.874) revealed that temperature was also the dominant driver of compositional variation within the five-species consortium, explaining 26.3% of the variation (*F*_1,35_ = 13.88, *p* = 0.0001; supplementary material, table S5). The main effect of pesticide was not statistically significant (2.2% of variation, *F*_1,35_ = 1.16, *p* = 0.314), and the temperature × pesticide interaction showed a marginal effect (4.8% of variation, *F*_1,35_ = 2.55, *p* = 0.068). NMDS ordination based on Bray–Curtis dissimilarities (figure 2A) primarily reflected changes in the relative abundances of the five focal taxa rather than emergent community structure. The primary compositional gradient along NMDS1 was strongly associated with temperature (environmental fit: *R*^2^ = 0.32, *p* = 0.0001), whereas the association with pesticide was negligible (*R*^2^ = 0.02, *p* = 0.400). Species vector overlays confirmed this pattern (all *R*^2^ ≥ 0.54, *p* ≤ 0.0001), in that both *Lactobacillus* species showed positive loadings on NMDS1 (≥ 0.68) and all *Acetobacter* negative loadings (≤–0.38; supplementary material, table S6), consistent with relatively greater abundance of *Acetobacter* under high-temperature conditions. Pesticide-associated variation was weaker and less directionally consistent, with *A*. *tropicalis* (–0.92) and *L. brevis* (–0.44) showing negative loadings on NMDS2 while the remaining species showed positive loadings (≥0.56), and the overall pesticide association with NMDS2 remained ambiguous.

**Figure 2.**
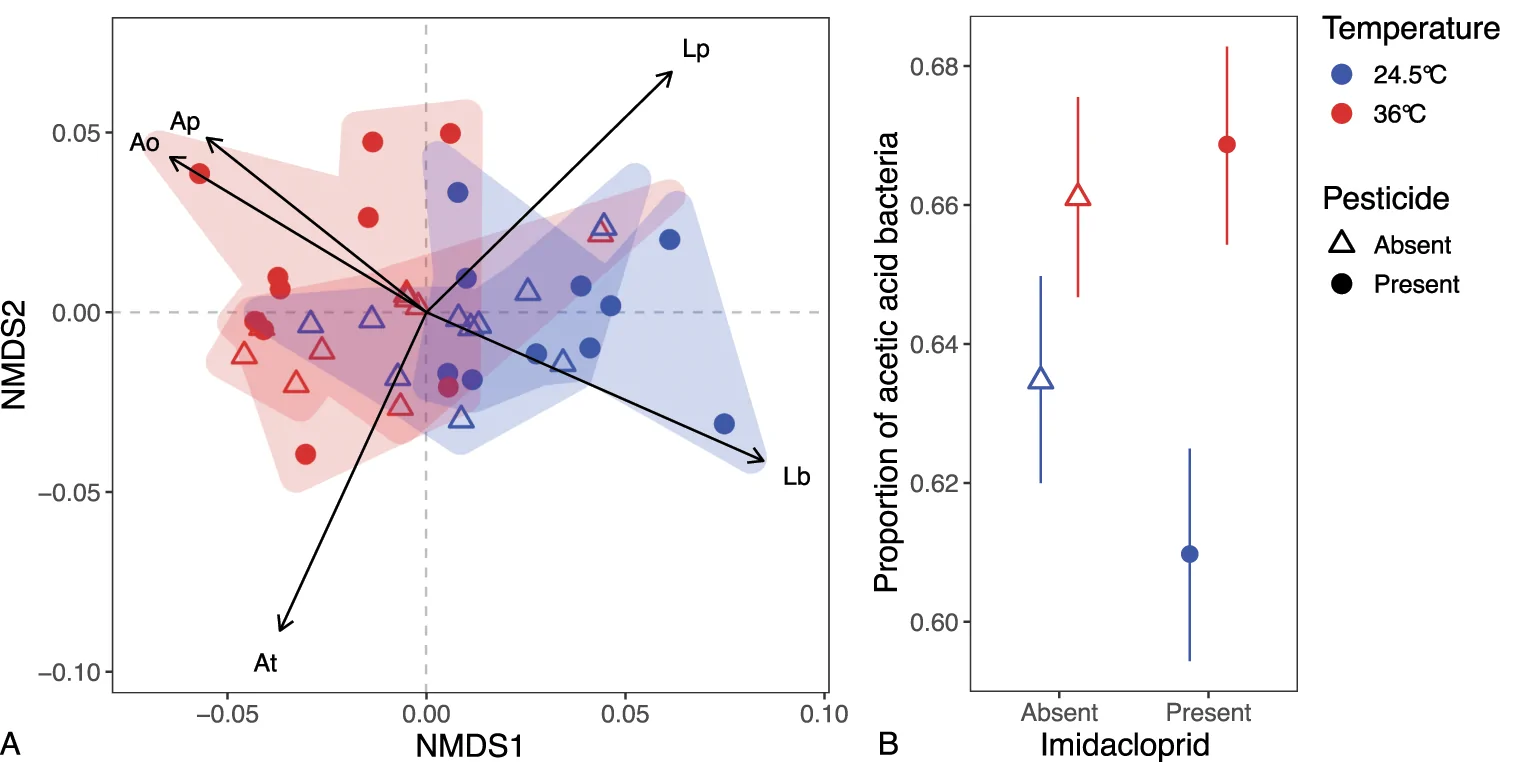
Bacterial abundances in the five-species consortium in response to thermal and pesticide treatments. (A) Non-metric multidimensional scaling (NMDS) ordination (Bray–Curtis distance, *k* = 4, stress = 0.046) of microbial community composition across temperature and pesticide treatments. Shaded convex hulls denote 95% confidence areas for each temperature × pesticide group, and arrows show the direction and strength of species (Hellinger-transformed) most strongly correlated with the ordination space, as identified by *envfit* (species: Ao, *Acetobacter orientalis*; Ap, *Acetobacter pasteurianus*; At, *Acetobacter tropicalis*; Lb, *Lactobacillus brevis*; Lp, *Lactobacillus plantarum*). (B) Proportion of acetic acid bacteria in the five-species consortium in response to heat and pesticide stress. Semi-transparent points represent the raw data, and solid symbols with error bars the model-predicted means and 95% confidence intervals.

Finally, in a binomial GLMM focusing only on the relative abundances of acetic acid versus lactic acid bacteria, where the three *Acetobacter* species should theoretically constitute 60% of the total CFUs among the five species, temperature and pesticide presence interacted significantly (*N* = 40, *z* = 2.15, *p* = 0.032). Specifically, the relative representation of *Acetobacter* was increased to around 66% under heat stress (*z* = 2.84, *p* = 0.013), whereas pesticide exposure reduced its representation (*z* = –2.32, *p* = 0.021), albeit only at the benign temperature (figure 3B).

**Figure 3.**
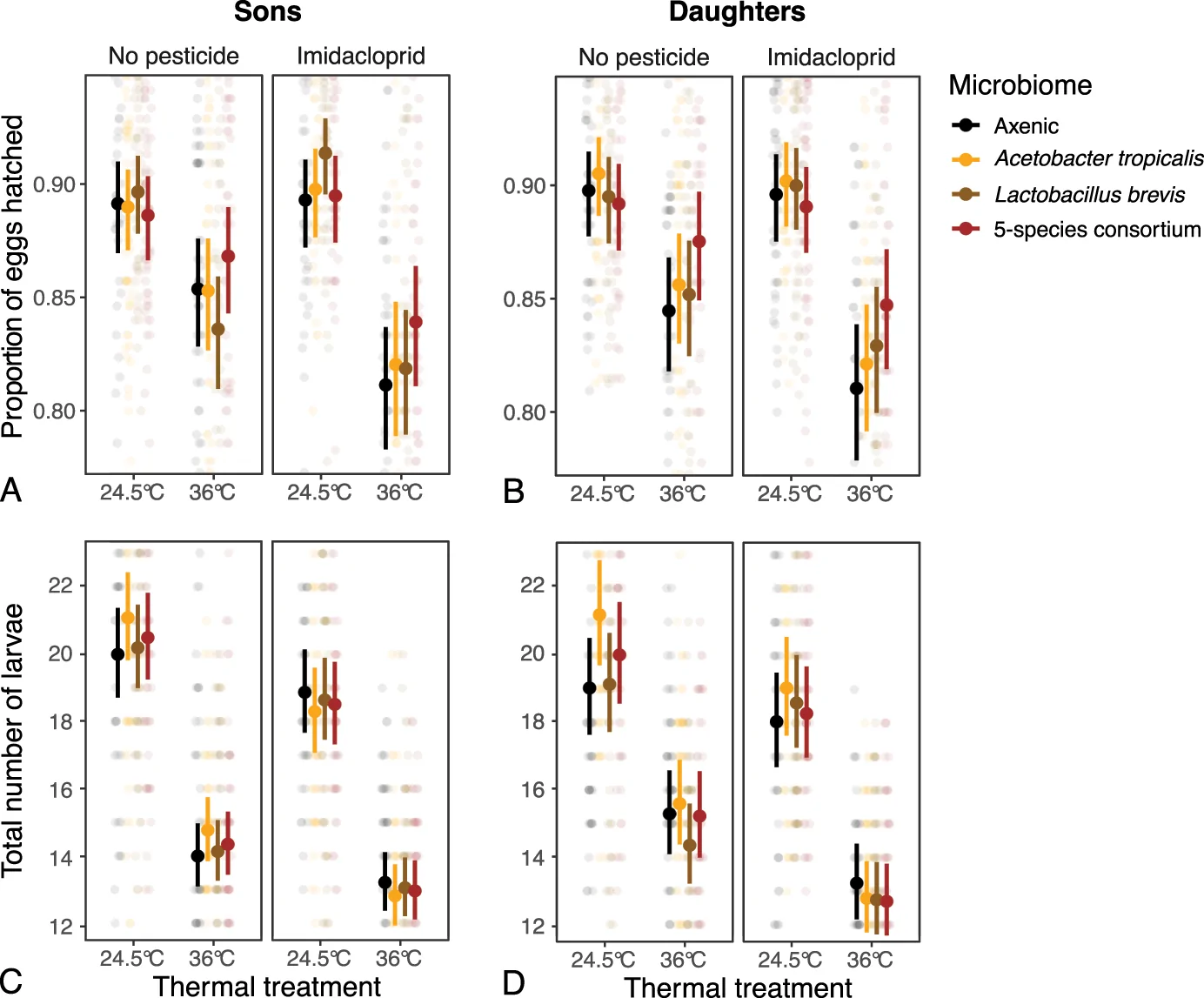
Fitness consequences of paternal thermal and pesticide treatments on fitness measures in male (A,C) and female (B,D) offspring. (A,B) Proportion of eggs hatched out of total number of eggs laid, (C,D) the fecundity either of the sons’ mates (C) or of the daughters (D). Semi-transparent points indicate the raw data, and solid points with error bars represent the model-predicted means and 95% confidence intervals.

### (e) Cross-generational effects on offspring fitness

Finally, we investigated how paternal stress exposure affects the next generation by measuring the hatching success and fecundity of F_1_ offspring produced by F_0_ males from each treatment group. We found a significant interactive effect between paternal temperature and pesticide treatments on the hatching success of both male (*N* = 725, *χ*² = 9.66, d.f. = 1, *p* = 0.002; supplementary material, table S7) and female offspring (*N* = 740, *χ*² = 4.68, d.f. = 1, *p* = 0.031; supplementary material, table S8; figure 3A,B), indicating that the effects of paternal stress exposure were transmitted to the next generation. However, the paternal microbiome contributed only to very weak and non-statistically significant trends for an interaction with temperature in both sons (*χ*² = 6.36, d.f. = 3, *p* = 0.10) and daughters (*χ*² = 6.11, d.f. = 3, *p* = 0.11), suggesting limited cross-generational effects on F_2_ hatching.

The interactive effect of paternal temperature and pesticide exposure was also significant for the fecundity of F_1_ daughters (*N* = 740, *χ*² = 6.06, d.f. = 1, *p* = 0.014), along with a main effect of the paternal microbiome composition (*χ*² = 8.99, d.f. = 3, *p* = 0.029; figure 3; supplementary material, table S9). By contrast, paternal effects on larval numbers in sons were limited to main effects of temperature and pesticide exposure (*χ*² ≥ 26.65, d.f. = 1, *p* < 0.0001), with no contribution of the paternal microbiome (*χ*² = 0.51, d.f. = 3, *p* = 0.918; figure 3; supplementary material, table S10).

## 4. Discussion

We investigated how the gut microbiome modulates male reproductive success in *Drosophila melanogaster* exposed to acute heat stress and the pesticide imidacloprid. Our results confirm previous reports that both sublethal temperatures [11,76–81] and imidacloprid [62,82–85] impair reproductive functions. While these stressors also acted synergistically in our experiments, the novel contributions of our work are twofold. First, we demonstrate that the gut microbiome mitigates the reproductive costs of combined heat and pesticide exposure, with *Acetobacter* generally providing stronger protection than *Lactobacillus,* especially in the presence of a more diverse microbial community. Second, we show that paternal stress exposure reduces offspring fitness, but these cross-generational effects were not mediated by the paternal microbiome under our experimental conditions which minimized direct microbial transmission. Together, these findings highlight the buffering role of microbial diversity against multiple environmental stressors and provide new insight into direct paternal effects on offspring fitness independent of microbial inheritance.

### (a) Interactive effects of multiple stressors

Our study revealed that initial exposure to imidacloprid followed by heat shock reduced male reproductive functions to a greater extent than either stressor alone, consistent with previous studies indicating complex, often order-dependent interactions between thermal stress and pesticide toxicity [17,86–88]. While pre-exposure to heat can sometimes increase insecticide tolerance [89–91], the reverse order often exacerbates toxicity [88,92]. Indeed, we found reduced reproductive success when pesticide exposure preceded heat stress, supporting studies that show higher imidacloprid toxicity at elevated temperatures in mayflies, earthworms, collembolans and *Hypogastrura viatica* [93–95]. These findings highlight that the sequence of exposure must be carefully considered when evaluating combined stressor effects on insect performance.

The mechanisms underlying these interactions likely involve compromised stress-response pathways or negative cross-talk between them, as well as resource trade-offs [96]. Coping with pesticide toxicity may deplete antioxidants, chaperones or energy reserves needed for thermal tolerance. Elevated temperature also increases maintenance costs [97], reducing resources for repair [98]. In addition, warmer conditions can enhance pesticide uptake and metabolism [99] and promote reactive oxygen species (ROS) accumulation [63,100]. Both heat and pesticide exposure induce heat-shock proteins [101–103], which may initially protect against stress but impose reproductive costs when overexpressed [104,105]. Moreover, combined exposure may also exacerbate neural dysfunction [23,30], potentially reducing mating performance, sperm transfer efficiency or ejaculate allocation, even in the absence of overt mortality [49–51]. These mechanisms explain why combined exposure produced stronger fitness losses than either stressor alone and emphasize the need to consider exposure sequence when evaluating temperature–toxicant interactions.

### (b) Microbiome as a modulator of resilience

A key finding of our study was the significant role of the gut microbiome in mitigating the detrimental reproductive consequences of heat and pesticide stress. Axenic males consistently performed worse than colonized males, showing a crucial role of microbes for reproductive fitness under stressful conditions. Interestingly, protection depended on microbial composition: *Acetobacter tropicalis* and *Lactobacillus brevis* offered partial buffering, while a five-species community provided the strongest resilience, emphasizing the potential importance of microbial diversity and inter-species interactions for robust host function [106–111]. Our results partially contrast with [112], which reported that *Lactobacillus plantarum* (Lp39) supplementation rescued *D. melanogaster* from harmful imidacloprid effects, whereas we did not observe such a strong rescue with *Lactobacillus brevis* in the present study. This discrepancy could stem from strain-specific functions, similar to opposing effects on chlorpyrifos (CP) toxicity between *L. plantarum* ISO (International Organization for Standardization) and *Lactobacillus* ATCC (American type Culture Collection) in *D. melanogaster* [112], or from differences in experimental protocols (e.g. our acute 18 h imidacloprid exposure versus Daisley *et al.*'s [112] continued exposure).

We also observed shifts in the relative abundance of *Acetobacter* and *Lactobacillus* within the host, aligning with studies showing heat-induced microbiome disruptions [32,33,113]. Generally, we found a higher proportional representation of *Acetobacter* following acute heat stress, consistent with prior work [33]. In *D. melanogaster*, gut microbes can directly modulate host physiological stress-response pathways, such as immune signalling or potentially Hsp expression in gut epithelium, and indirectly contribute to resilience through nutritional provisioning and metabolic regulation, including trehalose levels [104,114–116]. These functions are critical for thermal resilience and may explain why colonized males outperformed axenic ones under stress. Disruption of microbial partners can undermine these defences, as seen with heat-induced loss of *Buchnera aphidicola* reducing aphid heat tolerance [117] or temperature-driven microbiome shifts increasing imidacloprid susceptibility in planthoppers [118]. While our experimental design isolated the contribution of defined bacterial communities, yeasts also shape *Drosophila* nutrition and thermal performance (particularly cold tolerance; e.g. [119,120]) and represent an additional axis of host–microbiome resilience not captured here but worth pursuing in future studies. Similarly, although *Wolbachia* infection persisted after the axenization procedure, all flies underwent the same treatment before allocation to experimental groups. Consequently, the observed effects are attributable to differences in experimentally manipulated gut bacterial communities rather than variation in *Wolbachia* status. Future work could test whether interactions between gut bacteria and *Wolbachia* modify stress resilience under more natural microbiome compositions.

### (c) Microbiome composition and sexual selection dynamics

A central advance of our study is that we show how microbiome composition alters outcomes of sperm competition, a key component of post-mating sexual selection [121]. We found that *Acetobacter* and *Lactobacillus* differentially shaped sperm competition success (*P*_2_), while the multi-species consortium conferred the strongest resilience. Because *P*_2_ integrates multiple components of male performance—including mating success, ejaculate transfer and post-copulatory processes—it provides a particularly informative measure of reproductive fitness under stress, extending beyond pre-copulatory traits such as courtship or mating latency. These post-copulatory processes may also provide a mechanistic link to potential cross-generational effects via changes in sperm quality and ejaculate composition.

By shifting sperm competition dynamics, stress–microbiome interactions may change the strength and direction of sexual selection. Given sexual selection shapes the evolution of reproductive traits and population adaptability [122,123], these findings extend the role of microbiomes from buffering stress responses to influencing broader evolutionary trajectories. By integrating defined microbiomes, multi-stressor exposures and competitive mating assays, these findings extend the literature by showing that environmental stress and microbial composition do not just reduce fertility but actively reshape the outcomes of post-copulatory sexual selection in populations exposed to global change.

### (d) Cross-generational consequences

We also found clear evidence of cross-generational fitness costs, as paternal stress exposure reduced offspring fecundity and hatching success. These results align with previous reports of paternal heat or toxicant effects [124–126] and extend them by revealing synergistic impacts of combined stressors. Our findings support the view that cross-generational responses to environmental stress are often non-adaptive or costly and may contribute to cumulative fitness declines in populations exposed to recurrent multi-stressor events [127].

In contrast to the pronounced microbiome-mediated buffering observed within generations, we found little evidence that microbiome composition affected these cross-generational effects. This reveals a decoupling between within-generation resilience and cross-generational outcomes, whereby microbial benefits to paternal performance do not necessarily translate into protection for offspring. This pattern is consistent with our experimental design, which limited opportunities for microbial transmission across generations; females were axenic and maintained on sterile food, and both females and offspring were transferred to fresh sterile vials immediately after mating or hatching. These conditions strongly reduced diet-mediated microbial transmission, the dominant route of microbiome acquisition in laboratory *Drosophila*. While microbial transfer during copulation cannot be entirely excluded [128], such contributions are likely transient and unlikely to persist without environmental reinforcement. Consequently, the observed cross-generational effects are most consistent with direct paternal influences, such as gametic or epigenetic mechanisms. Future work that explicitly manipulates vertical transmission pathways, including maternal microbiome status and environmental exposure, will be essential to determine whether microbiomes can buffer or amplify cross-generational stress effects under more naturalistic conditions.

In addition, controlling microbiome composition likely reduced stochastic microbial heterogeneity among males, which is known to contribute substantially to variation in host physiology and reproductive performance in *Drosophila* [52,129,130]. By experimentally manipulating microbiomes to test their causal role in stress buffering, we also reduced an important source of uncontrolled environmental variance, thereby increasing precision in estimating microbiome × stress interactions.

## 5. Conclusions and broader implications

In conclusion, our study demonstrates that acute heat stress and sublethal pesticide exposure interact, often synergistically, to negatively impact male reproductive fitness in *D. melanogaster*, with consequences extending to offspring viability and fecundity. Beyond direct effects on fertility, our study reveals that microbiome–stressor interactions can also modulate post-mating sexual selection, with potential consequences for evolutionary trajectories and long-term population persistence. Crucially, we show that the gut microbiome is a key determinant of resilience, offering significant protection against these stressors. These benefits are further enhanced by microbial complexity, but the microbiome itself is vulnerable to disruption by these same stressors.

Together, our results identify the microbiome as a stress-sensitive but functionally protective axis of host resilience and show that multi-stressor interactions can amplify reproductive failure across generations. Given that heatwaves and pesticide exposure commonly co-occur, our data suggest that populations with simplified or imbalanced microbiomes may face increased risks of demographic decline. We recommend that ecological risk assessments explicitly incorporate (i) microbiome status, (ii) male-mediated, cross-generational effects and (iii) combined stressor exposure. Future research should explore these interactions across diverse species, environments and stressor combinations to improve ecological forecasting and develop strategies for mitigating biodiversity loss in the Anthropocene.

## Supplementary Material



## Data Availability

All data and R code supporting the findings of this study are publicly available in the Figshare Data Repository at [131]. Supplementary material is available online [132].
