# Supplementary material for: Microbiome-mediated resilience and cross-generational consequences in male Drosophila exposed to combined environmental stressors
Source: Proc Biol Sci. 2026 Oct 14;293(2080):20261300. doi: 10.1098/rspb.2026.1300 (PMC13678622; doi:10.1098/rspb.2026.1300)
Supplement: Supplementary file 1 [file rspb.2026.1300.zip › rspb.2026.1300/rspb20261300_si_001.pdf]

Supplementary material

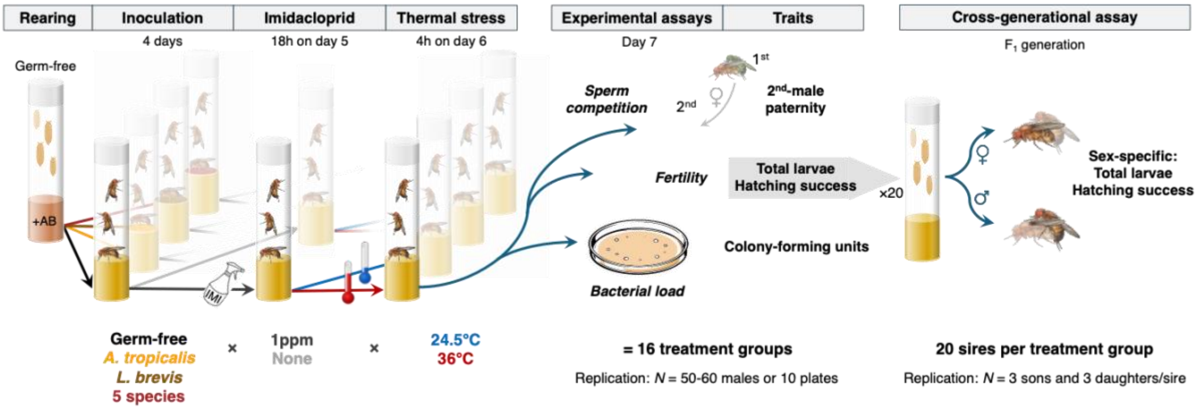

Figure S1: Experimental workflow, illustrating the full sequence from rearing axenic flies and bacterial inoculation to experimental treatments and assays in both the parental and filial generations. Initially, dechorionated eggs developed in sterile medium supplemented with a cocktail of antibiotics (+AB), whilst all remaining food was autoclaved but did not contain antibiotics (indicated by medium colour above). The four microbiome, two pesticide and two thermal treatments resulted in 16 treatment combinations, each of which was replicated 50–60 times across two blocks (total N = 800–960 trials), except for bacterial load that was based on 10 replicates across the same two blocks (total N = 160 plates, of which only the 120 colonised plates (non-axenic treatments) were considered for analysis). The fitness of 20 males per treatment group (10 from each block) of the non-competitive assay was followed through to the F_0 generation by quantifying fitness traits in three daughters and three sons per male (total N = 960 trials per sex). However, final sample sizes were reduced (see Tables S1–S10), primarily due to unsuccessful mating trials or lack of oviposition, but in a few isolated cases also due to mortality before oviposition was complete or accidental loss of flies during handling.

Table S1: Results from a generalized linear mixed-effects model on hatching success. The model assessed the impact of male microbiome (axenic, *Acetobacter tropicalis*, *Lactobacillus brevis*, 5 species), temperature (24.5°C/36°C), and pesticide exposure (absent/present). Total sample size: *N* = 650.

Predictor	*df*	χ^2	*P*
Microbiome	3	215.376	**<0.001**
Temperature	1	94.275	**<0.001**
Pesticide	1	67.431	**<0.001**
Microbiome × Temperature	3	21.888	**<0.001**
Microbiome × Pesticide	3	18.153	**<0.001**
Temperature × Pesticide	1	0.348	0.555
Microbiome × Temperature × Pesticide	3	14.968	**0.002**

Random effect.

Group	*Term*	*SD*	*Variance*
Block	(Intercept)	0.045	0.002

Fixed-effect coefficients.

Term	*Estimate*	*SE*	*z*	*P*
(Intercept)	0.944	0.085	11.123	**<0.001**
Microbiome[At]	1.498	0.141	10.602	**<0.001**
Microbiome[Lb]	0.856	0.133	6.450	**<0.001**
Microbiome[5 species]	1.960	0.154	12.712	**<0.001**
Temperature[36°C]	-1.080	0.111	-9.710	**<0.001**
Pesticide[Present]	-0.857	0.104	-8.212	**<0.001**
Microbiome[At] × Temperature[36°C]	-0.694	0.184	-3.775	**<0.001**
Microbiome[Lb] × Temperature[36°C]	-0.498	0.171	-2.905	**0.004**
Microbiome[5 species] × Temperature[36°C]	-0.717	0.189	-3.794	**<0.001**
Microbiome[At] × Pesticide[Present]	-0.701	0.173	-4.045	**<0.001**
Microbiome[Lb] × Pesticide[Present]	-0.355	0.166	-2.140	**0.032**
Microbiome[5 species] × Pesticide[Present]	-0.482	0.186	-2.583	**0.010**
Temperature[36°C] × Pesticide[Present]	0.089	0.151	0.590	**0.555**
Microbiome[At] × Temperature[36°C] × Pesticide[Present]	0.871	0.236	3.694	**<0.001**
Microbiome[Lb] × Temperature[36°C] × Pesticide[Present]	0.534	0.226	2.363	**0.018**
Microbiome[5 species] × Temperature[36°C] × Pesticide[Present]	0.574	0.239	2.400	**0.016**

Table S2: Results from a generalized linear mixed-effects model on the total number of larvae produced. The model assessed the impact of male microbiome (axenic, *Acetobacter tropicalis*, *Lactobacillus brevis*, 5 species), temperature (24.5°C/36°C), and pesticide exposure (absent/present). The three-way interaction was removed due to non-significance ($P = 0.88$). Total sample size: $N = 650$.

Predictor	*df*	χ^2	*P*
Microbiome	3	207.454	**<0.001**
Temperature	1	85.347	**<0.001**
Pesticide	1	23.944	**<0.001**
Microbiome × Temperature	3	8.057	**0.045**
Microbiome × Pesticide	3	4.409	0.234
Temperature × Pesticide	1	7.842	**0.005**

Random effect.

Group	*Term*	*SD*	*Variance*
Block	(Intercept)	0.000	0.000

Fixed-effect coefficients.

Term	*Estimate*	*SE*	*z*	*P*
(Intercept)	2.564	0.039	66.243	**<0.001**
Microbiome[At]	0.442	0.049	9.019	**<0.001**
Microbiome[Lb]	0.277	0.053	5.237	**<0.001**
Microbiome[5 species]	0.652	0.047	13.877	**<0.001**
Temperature[36°C]	-0.522	0.057	-9.238	**<0.001**
Pesticide[Present]	-0.260	0.053	-4.893	**<0.001**
Microbiome[At] × Temperature[36°C]	0.151	0.069	2.171	**0.030**
Microbiome[Lb] × Temperature[36°C]	0.092	0.072	1.287	0.198
Microbiome[5 species] × Temperature[36°C]	0.173	0.064	2.692	**0.007**
Microbiome[At] × Pesticide[Present]	0.021	0.066	0.318	0.751
Microbiome[Lb] × Pesticide[Present]	-0.056	0.069	-0.816	0.414
Microbiome[5 species] × Pesticide[Present]	0.065	0.062	1.045	0.296
Temperature[36°C] × Pesticide[Present]	-0.125	0.044	-2.800	**0.005**

Table S3: Results from a generalized linear mixed-effects model on the proportion of second-male offspring (sperm offense ability). The model assessed the impact of male microbiome (axenic, *Acetobacter tropicalis*, *Lactobacillus brevis*, 5 species), temperature (24.5°C/36°C), and pesticide exposure (absent/present). All interactions were sequentially removed due to non-significance ($P > 0.15$). Total sample size: $N = 331$.

Predictor	*df*	χ^2	*P*
Microbiome	3	219.226	**<0.001**
Temperature	1	162.650	**<0.001**
Pesticide	1	101.937	**<0.001**

Random effect.

Group	*Term*	*SD*	*Variance*
Block	(Intercept)	0.000	0.000

Fixed-effect coefficients.

Term	*Estimate*	*SE*	*z*	*P*
(Intercept)	0.422	0.062	6.847	**<0.001**
Microbiome[At]	0.703	0.068	10.365	**<0.001**
Microbiome[Lb]	0.357	0.068	5.279	**<0.001**
Microbiome[5 species]	0.987	0.072	13.736	**<0.001**
Temperature[36°C]	-0.604	0.047	-12.753	**<0.001**
Pesticide[Present]	-0.480	0.048	-10.096	**<0.001**

Table S4: Results from a linear mixed-effects model on colony forming units. The model assessed the impact of male microbiome (axenic, *Acetobacter tropicalis*, *Lactobacillus brevis*, 5 species), temperature (24.5°C/36°C), and pesticide exposure (absent/present). Total sample size: N = 120.

Predictor	*df*	χ^2	*P*
Temperature	1	137.764	**<0.001**
Pesticide	1	158.883	**<0.001**
Microbiome	2	2,015.292	**<0.001**
Temperature × Pesticide	1	12.102	**0.001**
Temperature × Microbiome	2	21.421	**<0.001**
Pesticide × Microbiome	2	91.646	**<0.001**
Temperature × Pesticide × Microbiome	2	10.128	**0.006**

Random effects.

Group	*Term*	*SD*	*Variance*
Block	(Intercept)	0.000	0.000
Residual	(Intercept)	0.029	0.001

Coefficients of the model described above.

Term	*Estimate*	*SE*	*t*	*P*
(Intercept)	4.532	0.009	493.900	**<0.001**
Temperature[36°C]	-0.152	0.013	-11.737	**<0.001**
Pesticide[Present]	-0.164	0.013	-12.605	**<0.001**
Microbiome[Lb]	0.166	0.013	12.807	**<0.001**
Microbiome[5 species]	0.567	0.013	43.665	**<0.001**
Temperature[36°C] × Pesticide[Present]	0.064	0.018	3.479	**<0.001**
Temperature[36°C] × Microbiome[Lb]	0.030	0.018	1.637	0.105
Temperature[36°C] × Microbiome[5 species]	0.084	0.018	4.568	**<0.001**
Pesticide[Present] × Microbiome[Lb]	0.131	0.018	7.143	**<0.001**
Pesticide[Present] × Microbiome[5 species]	0.167	0.018	9.091	**<0.001**
Temperature[36°C] × Pesticide[Present] × Microbiome[Lb]	-0.068	0.026	-2.630	**0.010**
Temperature[36°C] × Pesticide[Present] × Microbiome[5 species]	-0.074	0.026	-2.867	**0.005**

Table S5: Results of a PERMANOVA examining the temperature and pesticide effects on relative bacterial abundances (based on colony-forming units) in the five-species consortium.

Predictor	*df*	*Sum of squares*	R^2	*F*	*P*
Temperature	1	0.004	0.263	13.882	**<0.001**
Pesticide	1	<0.001	0.022	1.157	0.314
Block	1	<0.001	0.004	0.202	0.874
Temperature × Pesticide	1	<0.001	0.048	2.553	*0.068*
Residual	35	0.011	0.663		
Total	39	0.017	1.000		

Table S6: Ordination of the Bray-Curtis dissimilarities between the relative abundances of the five bacterial species via non-metric multidimensional scaling (NMDS).

Species	*NMDS1*	*NMDS2*	R^2	*P*
A. tropicalis	-0.384	-0.923	0.920	**<0.001**
A. orientalis	-0.831	0.556	0.601	**<0.001**
A. pasteurianus	-0.751	0.660	0.539	**<0.001**
L. brevis	0.899	-0.439	0.887	**<0.001**
L. plantarum	0.678	0.735	0.826	**<0.001**

Table S7: Results from a binomial generalized linear mixed-effects model on hatching success among the eggs laid by the mates of the sons. The model assessed the impact of male microbiome (axenic, *Acetobacter tropicalis*, *Lactobacillus brevis*, 5 species), temperature (24.5°C/36°C), and pesticide exposure (absent/present), incorporating sire to account for non-independence among siblings. The three-way interaction was removed due to non-significance ($P = 0.48$). Total sample size: $N = 725$.

Predictor	*df*	χ^2	*P*
Microbiome	3	0.832	0.842
Temperature	1	8.488	**0.004**
Pesticide	1	0.019	0.890
Microbiome × Temperature	3	6.355	*0.096*
Microbiome × Pesticide	3	1.708	0.635
Temperature × Pesticide	1	9.659	**0.002**

Random effects.

Group	*Term*	*SD*	*Variance*
Sire	(Intercept)	0.000	0.000
Block	(Intercept)	0.051	0.003

Fixed-effect coefficients.

Term	*Estimate*	*SE*	*z*	*P*
(Intercept)	2.103	0.106	19.821	**<0.001**
Microbiome[At]	-0.015	0.126	-0.122	0.903
Microbiome[Lb]	0.054	0.127	0.429	0.668
Microbiome[5 species]	-0.052	0.127	-0.410	0.682
Temperature[36°C]	-0.340	0.117	-2.913	**0.004**
Pesticide[Present]	0.016	0.118	0.139	0.890
Microbiome[At] × Temperature[36°C]	0.009	0.147	0.064	0.949
Microbiome[Lb] × Temperature[36°C]	-0.190	0.143	-1.322	0.186
Microbiome[5 species] × Temperature[36°C]	0.173	0.146	1.179	0.238
Microbiome[At] × Pesticide[Present]	0.066	0.146	0.448	0.654
Microbiome[Lb] × Pesticide[Present]	0.183	0.142	1.285	0.199
Microbiome[5 species] × Pesticide[Present]	0.072	0.146	0.494	0.621
Temperature[36°C] × Pesticide[Present]	-0.321	0.103	-3.108	**0.002**

Table S8: Results from a binomial generalized linear mixed-effects model on hatching success among the eggs laid by the daughters. The model assessed the impact of male microbiome (axenic, *Acetobacter tropicalis*, *Lactobacillus brevis*, 5 species), temperature (24.5°C/36°C), and pesticide exposure (absent/present), incorporating sire to account for non-independence among siblings. The three-way interaction was removed due to non-significance (P = 0.87). Total sample size: N = 740.

Predictor	*df*	χ^2	*P*
Microbiome	3	1.507	0.681
Temperature	1	17.827	**<0.001**
Pesticide	1	0.025	0.875
Microbiome × Temperature	3	6.110	0.106
Microbiome × Pesticide	3	0.445	0.931
Temperature × Pesticide	1	4.679	**0.031**

Random effects.

Group	*Term*	*SD*	*Variance*
Sire	(Intercept)	0.000	0.000
Block	(Intercept)	0.062	0.004

Fixed-effect coefficients.

Term	*Estimate*	*SE*	*z*	*P*
(Intercept)	2.170	0.104	20.955	**<0.001**
Microbiome[At]	0.084	0.126	0.666	0.505
Microbiome[Lb]	-0.029	0.127	-0.229	0.819
Microbiome[5 species]	-0.062	0.126	-0.496	0.620
Temperature[36°C]	-0.478	0.113	-4.222	**<0.001**
Pesticide[Present]	-0.018	0.116	-0.157	0.875
Microbiome[At] × Temperature[36°C]	0.006	0.144	0.043	0.966
Microbiome[Lb] × Temperature[36°C]	0.085	0.144	0.591	0.555
Microbiome[5 species] × Temperature[36°C]	0.316	0.146	2.168	**0.030**
Microbiome[At] × Pesticide[Present]	-0.019	0.143	-0.133	0.894
Microbiome[Lb] × Pesticide[Present]	0.071	0.144	0.494	0.621
Microbiome[5 species] × Pesticide[Present]	0.005	0.146	0.037	0.970
Temperature[36°C] × Pesticide[Present]	-0.222	0.103	-2.163	**0.031**

Table S9: Results from a Poisson generalized linear mixed-effects model on the total number of larvae produced by the daughters. The model assessed the impact of male microbiome (axenic, *Acetobacter tropicalis*, *Lactobacillus brevis*, 5 species), temperature (24.5°C/36°C), and pesticide exposure (absent/present), incorporating sire to account for non-independence among siblings. The three-way interaction was removed due to non-significance ($P = 0.22$). Total sample size: $N = 740$.

Predictor	*df*	χ^2	*P*
Microbiome	3	8.986	**0.029**
Temperature	1	28.908	**<0.001**
Pesticide	1	1.797	0.180
Microbiome × Temperature	3	3.221	0.359
Microbiome × Pesticide	3	2.955	0.399
Temperature × Pesticide	1	6.063	**0.014**

Random effects.

Group	*Term*	*SD*	*Variance*
Sire	(Intercept)	0.000	0.000
Block	(Intercept)	0.033	0.001

Fixed-effect coefficients.

Term	*Estimate*	*SE*	*z*	*P*
(Intercept)	2.946	0.039	76.480	**<0.001**
Microbiome[At]	0.109	0.041	2.645	**0.008**
Microbiome[Lb]	0.006	0.042	0.139	0.889
Microbiome[5 species]	0.051	0.042	1.212	0.226
Temperature[36°C]	-0.219	0.041	-5.377	**<0.001**
Pesticide[Present]	-0.053	0.040	-1.341	0.180
Microbiome[At] × Temperature[36°C]	-0.089	0.052	-1.720	*0.085*
Microbiome[Lb] × Temperature[36°C]	-0.069	0.052	-1.312	0.189
Microbiome[5 species] × Temperature[36°C]	-0.055	0.052	-1.050	0.294
Microbiome[At] × Pesticide[Present]	-0.054	0.051	-1.064	0.287
Microbiome[Lb] × Pesticide[Present]	0.025	0.052	0.476	0.634
Microbiome[5 species] × Pesticide[Present]	-0.037	0.051	-0.727	0.467
Temperature[36°C] × Pesticide[Present]	-0.091	0.037	-2.462	**0.014**

Table S10: Results from a Poisson generalized linear mixed-effects model on the total number of larvae produced by the mates of the sons. The model assessed the impact of male microbiome (axenic, *Acetobacter tropicalis*, *Lactobacillus brevis*, 5 species), temperature (24.5°C/36°C), and pesticide exposure (absent/present), incorporating sire to account for non-independence among siblings. The interactions were sequentially removed due to non-significance ($P \geq 0.19$). Total sample size: $N = 725$.

Predictor	*df*	χ^2	*P*
Microbiome	3	0.505	0.918
Temperature	1	369.212	**<0.001**
Pesticide	1	26.653	**<0.001**

Random effects.

Group	*Term*	*SD*	*Variance*
Sire	(Intercept)	0.000	0.000
Block	(Intercept)	0.028	0.001

Fixed-effect coefficients.

Term	*Estimate*	*SE*	*z*	*P*
(Intercept)	3.014	0.030	100.771	**<0.001**
Microbiome[At]	0.015	0.026	0.558	0.577
Microbiome[Lb]	-0.002	0.026	-0.083	0.934
Microbiome[5 species]	0.003	0.026	0.104	0.917
Temperature[36°C]	-0.357	0.019	-19.215	**<0.001**
Pesticide[Present]	-0.095	0.018	-5.163	**<0.001**

Materials and Methods

Experimental populations

Throughout this study, we used an outbred *D. melanogaster* population (LH_m wild-type strain), maintained under constant conditions of 25°C, 60% humidity, and a 12h:12h light:dark cycle. First, we generated axenic (germ-free) focal flies by collecting eggs (within six hours of oviposition) from four large population containers supplied with Petri dishes containing grape-juice agar medium (300 mL water, 670 mL grape juice, 22 g agar, 20 g sugar, 19 g yeast, 20 mL nipagin per litre) supplemented with a smear of yeast paste. Eggs were washed off the plates into a strainer, pooled, and dechorionated by immersion in 3.5% bleach for 2 minutes, followed by a rinse in 70% ethanol and three rinses in sterile water.

Next, eggs were aseptically transferred to sterile glass vials containing autoclaved standard fly food (75 g glucose, 100 g fresh yeast, 55 g corn, 10 g flour, and 15 mL nipagin antimicrobial agent per litre food media) supplemented with antibiotics [a cocktail of following antibiotics with their respective final concentrations: Ampicillin (50 µg/mL), Kanamycin (50 µg/mL), Erythromycin (10 µg/mL), Tetracycline (10 µg/mL)]. Vials were then sealed with sterile plugs and incubated under standard laboratory conditions until adult eclosion. All steps of vial preparation were conducted in a laminar flow chamber to avoid contamination.

Upon eclosion, axenic status was confirmed using two methods on a subsample of flies. First, homogenized flies were plated on Lysogeny Broth (LB) and de Man, Rogosa and Sharpe (MRS) agar plates, incubated, and checked for bacterial colony growth. Second, genomic DNA was extracted from pools of 3-5 putatively germ-free and conventional flies (positive control), respectively, using Quick-DNA midiprep plus kit following the instructions provided by the manufacturer. Universal 16S primers (27F and 1492R) were used as forward and reverse primer, respectively. To prepare the PCR master mix, 10 µL of nuclease-free water, 0.5 µL of forward and reverse primer, 1µL of template DNA and 13 µL Taq green master mix were mixed in a sterile Eppendorf tube. PCR conditions were: initial denaturation at 95°C for 90s, followed by 35 cycles of denaturation at 95°C for 30s, annealing at 55°C for 30s and extension at 72°C for 90s, and final extension at 72°C for 5 minutes). PCR products were visualized on a 1% agarose gel under ultraviolet light to confirm the absence of bacterial DNA amplification in axenic samples and its presence in controls. No amplification was detected in axenic individuals, confirming the absence of detectable bacterial DNA. This also applied to the endosymbiont *Wolbachia*, which has been reported to affect the commensal microbiome composition [1] and thermal preference of its host (e.g., [2,3]). The lack of detectable amplification of both 16S and wsp (*Wolbachia* surface protein) suggests that tetracyclin in the antibiotic cocktail also eliminated *Wolbachia*.

Bacterial cultures and gnotobiotic associations

Axenic males were associated with defined compositions of microbial species, all originally isolated from the gut of wild-type flies [4]: *Acetobacter tropicalis* (*At*), and *Lactobacillus brevis* (*Lb*) for mono-associations based on their contrasting effects on male reproductive investments in our previous studies [5]. For comparison, we also included axenic males and males inoculated with a defined five-species community consisting of common and abundant *Drosophila* gut bacteria (*A. tropicalis*, *A. orientalis, A. pasteurianus, L. plantarum, L. brevis*), resulting in four distinct microbial treatments.

Bacterial strains were cultured overnight in an incubator at 30°C: *Acetobacter* in MRS + mannitol medium with shaking, and *Lactobacillus* in MRS broth under stationary conditions. The following

morning, cultures were checked for growth before measuring their optical densities (OD) at 600 nm and centrifuging at 7000 rpm for 5 minutes. Bacterial pellets were then resuspended in sterile phosphate-buffered saline (PBS) to a final concentration of 10^8 cells/mL, following established protocols [6,7].

To inoculate vials, 50µL of bacterial suspension (approx. 5×10^6 CFUs) was added to sterile food vials, with equal representation of bacterial strains in the 5-species consortia. This bacterial density is comparable to previous reports for conventionally reared flies [7,8]. Inoculated food vials were then allowed to air-dry in a laminar flow cell before introducing ten 1-day-old axenic males to each to generate gnotobiotic flies. Males were transferred to freshly inoculated vials with the same bacterial treatment every 48h for a total of four days to ensure stable colonization. Successful colonization with the intended bacteria was confirmed by plating homogenates of males from each treatment and checking for characteristic colony morphologies (see *Bacterial abundance of males*).

Stress treatments

Following the 4-day colonization period, males were transferred to vials containing autoclaved food (without antibiotics) and exposed to pesticide and heat stress in a fully factorial design. Specifically, half of the males from each microbial group were subjected to food supplemented with imidacloprid, whilst the remaining half were maintained on uncontaminated standard food. We used a concentration of 1 ppm (parts per million) of imidacloprid as concentrations of 0.04 to 20 ppm have been used effectively in studies on larval or adult *D. melanogaster*, depending on the exposure regime [9–11]. These concentrations are low enough to avoid immediate lethality but sufficient to induce sublethal effects on behaviour, reproduction, and physiology. Males were exposed to imidacloprid for an acute window of 18 h, following an additional thermal treatment of 4-hour in a hot water bath. To this end, half of the males from each microbiome and pesticide treatment were subjected to acute heat stress by transferring them to vials containing only sterile 1% agar (to prevent dehydration) and placing these vials in a water at 36°C for 4 hours. The remaining males (thermal controls) were treated identically but at benign 24.5°C. All males were then allowed to recover overnight in vials on fresh, autoclaved standard food under standard laboratory conditions (see *Experimental populations*).

Hatching success and fertility

To quantify the fitness consequences of heat and pesticide exposure and their potential mitigation by the microbiome on the morning after the stress treatments, experimental males were individually mated to standard females of the same population. All experimental males, regardless of microbiome or stress treatment, were mated with virgin axenic females derived from the same standardized cohort. Females were maintained under identical rearing conditions and were not subjected to microbial or stress treatments. Using a uniform female background ensured that variation in reproductive output primarily reflected male treatment effects rather than differences in female microbiome status or physiological condition.

After mating ended, females were allowed to lay eggs for 24 h, and another 30 h later (allowing time for embryonic development), oviposition vials were frozen for later quantification of the proportion of eggs hatching as a proxy of fertilization success, and the total number of larvae estimating male-mediated fecundity. This assay was performed in two blocks with 25 males per treatment combination in each, totalling 800 experimental males (4 bacterial treatments × 2 pesticide treatments × 2 temperature treatments × 2 blocks × 25 males). However, some vials were excluded due to failure to mate within the observation period, female mortality before the end of the oviposition period, zero egg production, or occasional loss of individuals during handling (e.g., escape during transfer). The final dataset used for analysis comprised 650 observations.

Sperm competitiveness

To assess male sperm competitiveness, the above experimental design was repeated, but this time using standard females that had mated once with standard competitor males two days before the focal mating. These standard competitors were of the same strain but genetically modified to ubiquitously express green fluorescent protein (GFP), permitting paternity assignment against the focal wild-type males [12,13]. Pairs were monitored for remating for up to 8 h. Remated females were then isolated in fresh vials for oviposition for two days, while pairs not mating were separated and given another 8-hour mating opportunity on the following day. Females still not remating were excluded. Oviposition vials were incubated until adult emergence to determine the paternity (proportion of focal-male offspring) based on the GFP expression of first-male offspring under a fluorescence stereoscope. The treatment design of this assay was identical to the non-competitive experiment above, but this time with 30 trials per treatment combination in each of the two experimental blocks (i.e., 960 trials in total).

The restricted remating window of 8 h on days two and three after the females' first mating was chosen to standardize microbial exposure across males. Because focal males were maintained on sterile food during the mating assays, extending trials over additional days risked progressive microbial depletion and uncontrolled variation in bacterial abundance among individuals. The assay therefore employed a tightly standardized exposure window rather than extending remating opportunities. Given the reduced remating propensity of axenic females in our previous work [5], we initiated 30 trials per treatment combination in each block to ensure adequate final replication. However, realized remating success was lower than anticipated, and additional attrition from insufficient offspring production (< 5 offspring available for paternity analysis), mortality, or handling losses contributed to the final sample size of 331 trials. Importantly, attrition was distributed approximately evenly across treatment combinations (final range: 17–25 replicates per cell), suggesting that the reduced sample size did not introduce directional bias into P_2 estimates.

Bacterial abundance of males

To investigate the impact of stress treatments on the bacterial load, colony forming units (CFUs) were compared between ten random males from each experimental treatment, surface-sterilized with 70% ethanol, and individually homogenized in 1.5 mL microcentrifuge tubes containing sterile PBS using a pestle. Homogenates were serially diluted to different dilutions at a final volume of 100 µL, then plated on MRS media for quantification of CFUs at appropriate dilutions (approx. 100 CFUs/plate). For the groups with both *Acetobacter* and *Lactobacillus* species (5-species consortium), MRS agar was supplemented with 10 µg/mL ampicillin to avoid the growth of *Lactobacillus,* whilst replicate plates were incubated in a CO_2 atmosphere to exclude the growth of *Acetobacter*. The presence of only target bacterial species on plates was confirmed based on colony morphologies, with *A. tropicalis* producing small tan-brown colonies and *L. brevis* producing small yellowish colonies. CFUs per fly were calculated as: $CFU = C \times D/P \times V$, where C = number of colonies counted, D = dilution, P = volume plated, V = volume of fly homogenate [6]. For males harbouring all the five bacterial species, bacterial colonies were identified by their morphology and total CFUs per fly was obtained by adding CFUs from all the five bacterial species.

Cross-generational effects

To assess cross-generational impacts of paternal (F_0) stress exposure, we quantified hatching success and fecundity in both male and female offspring (F_1) of our experimental males. Within each experimental block, up to 20 freshly hatched larvae from each of ten randomly selected vials per parental treatment combination (i.e., 16 combinations) were transferred to rearing vials containing autoclaved standard fly food. This immediate transfer minimized the potential acquisition of parental

microbes via consumption of the egg chorion or contaminated food. These larvae developed under standard laboratory conditions (see *Experimental population*).

Upon eclosion, 4-5 virgin F_1 male and female offspring were collected from each rearing vial and separated by sex. At 2-3 days of age, three males and three females from each lineage were individually mated with a virgin standard fly of the opposite sex, reared under axenic conditions (to avoid potential confounding effects of their microbiota). Following copulation, hatching success and fecundity were measured for these F_1 flies as in their parents (see above). These offspring themselves were not subjected to any experimental treatment, thus assessing solely the cross-generational effects. This experiment was carried out in two independent blocks of 480 offspring per sex from 160 F_0 males (i.e. total of 1,920 mating trials), but the final datasets were reduced to 725 sons and 740 daughters due to lack of eggs and the inability to unequivocally attribute any infertility to focal rather than standard flies.

Statistical analyses

All statistical analyses were performed in R version 4.4.3 [14], using generalized linear mixed-effects models (GLMMs) with a Poisson (fecundity) or a binomial error distribution (hatching success, paternity shares), or using a linear mixed-effects model on the log scale in case of bacterial abundance (CFUs). Each model included microbiome composition (4-level factor) along with the binary thermal and pesticide treatments with all two- and three-way interactions as fixed effects, whilst experimental block and, in the filial generation, sire were the random effects.

The binomial model of the relative abundance of *Acetobacter* versus *Lactobacillus* exhibited slight overdispersion (dispersion factor = 1.12), which we addressed by including an observation-level random effect. In contrast, the remaining non-linear models showed moderate underdispersion (dispersion factors ≈ 0.9). Alternative model families (e.g., negative binomial) did not improve model fit, so the original models were retained for clarity and interpretability. Throughout, model performance was checked using the performance package [15], and output plots were generated was plotted using ggplot2 [16] based on means and 95% confidence intervals estimated via *emmeans* [17].

The impact of the different experimental treatments was also analysed for the total bacterial abundances (CFUs) and, separately, for compositional shifts in the five-species consortia. For the former, we used a linear mixed-effects model with block as a random effect. For microbiome community composition in the five-species consortium, we quantified the species-specific abundances per sample, then applied a Hellinger transformation to these count data and computed Bray-Curtis distances using the *vegdist* function in the package vegan [18]. We assessed treatment effects (temperature, pesticide, and their interaction) in a permutational multivariate analysis of variance (PERMANOVA) using *adonis2* in the same package. To visualize compositional shifts, we further ran non-metric multidimensional scaling (NMDS) (*metaMDS*) on the Bray-Curtis dissimilarities and fitted species vectors onto the NMDS ordination with *envfit*, which indicates the direction and strength of each species-specific association with the ordination space. We also compared the relative abundances at the genus level, using a binomial GLMM with block as a random effect and accounting for overdispersion by including an additional observation-level random effect.

References

1. Simhadri RK, Fast EM, Guo R, Schultz MJ, Vaisman N, Ortiz L, Bybee J, Slatko BE, Frydman HM. 2017 The gut commensal microbiome of *Drosophila melanogaster* is modified by the endosymbiont *Wolbachia*. *mSphere* **2**, e00287-17. (doi:10.1128/mSphere.00287-17)

2. Truitt AM, Kapun M, Kaur R, Miller WJ. 2018 *Wolbachia* modifies thermal preference in *Drosophila melanogaster*. *Environ. Microbiol.* **21**, 3259–3268. (doi:10.1111/1462-2920.14347)

3. Strunov A, Schönherr C, Kapun M. 2024 *Wolbachia* effects on thermal preference of natural *Drosophila melanogaster* are influenced by host genetic background, *Wolbachia* type, and bacterial titer. *Environ. Microbiol.* **26**, e16579. (doi:10.1111/1462-2920.16579)

4. Obadia B, Güvener ZT, Zhang V, Ceja-Navarro JA, Brodie EL, Ja WW, Ludington WB. 2017 Probabilistic invasion underlies natural gut microbiome stability. *Curr. Biol.* **27**, 1999-2006.e8. (doi:10.1016/j.cub.2017.05.034)

5. Maggu K, Meena A, De Nardo AN, Sbilordo SH, Roy J, Lüpold S. 2025 Male gut microbiome mediates postmating sexual selection in *Drosophila*. *bioRxiv* (doi:10.1101/2025.05.23.655737)

6. Koyle ML, Veloz M, Judd AM, Wong AC-N, Newell PD, Douglas AE, Chaston JM. 2016 Rearing the fruit fly *Drosophila melanogaster* under axenic and gnotobiotic conditions. *J. Vis. Exp.* **113**, e54219. (doi:10.3791/54219)

7. Newell PD, Douglas AE. 2014 Interspecies interactions determine the impact of the gut microbiota on nutrient allocation in *Drosophila melanogaster*. *Appl. Environ. Microbiol.* **80**, 788–796. (doi:10.1128/AEM.02742-13)

8. Gould AL *et al.* 2018 Microbiome interactions shape host fitness. *Proc. Natl. Acad. Sci. U. S. A.* **115**, E11951–E11960. (doi:10.1073/pnas.1809349115)

9. Charpentier G, Louat F, Bonmatin J-M, Marchand PA, Vanier F, Locker D, Decoville M. 2014 Lethal and sublethal effects of imidacloprid, after chronic exposure, on the insect model *Drosophila melanogaster*. *Environ. Sci. Technol.* **48**, 4096–4102. (doi:10.1021/es405331c)

10. Frantzios G, Paptsiki K, Sidiropoulou B, Lazaridis I, Theophilidis G, Mavragani-Tsipidou P. 2008 Evaluation of insecticidal and genotoxic effects of imidacloprid and acetochlor in *Drosophila melanogaster*. *J. Appl. Entomol.* **132**, 583–590. (doi:10.1111/j.1439-0418.2008.01269.x)

11. Martelli F *et al.* 2020 Low doses of the neonicotinoid insecticide imidacloprid induce ROS triggering neurological and metabolic impairments in *Drosophila*. *Proc. Natl. Acad. Sci.* **117**, 25840–25850. (doi:10.1073/pnas.2011828117)

12. Lüpold S, Manier MK, Berben KS, Smith KJ, Daley BD, Buckley SH, Belote JM, Pitnick S. 2012 How multivariate ejaculate traits determine competitive fertilization success in *Drosophila melanogaster*. *Curr. Biol.* **22**, 1667–1672. (doi:10.1016/j.cub.2012.06.059)

13. Lüpold S, Reil JB, Manier MK, Zeender V, Belote JM, Pitnick S. 2020 How female × male and male × male interactions influence competitive fertilization in *Drosophila melanogaster*. *Evol. Lett.* **4**, 416–429. (doi:10.1002/evl3.193)

14. R Core Team. 2025 *R: A language and environment for statistical computing*. Vienna, Austria: R Foundation for Statistical Computing.

15. Lüdecke D, Ben-Shachar MS, Patil I, Waggoner P, Makowski D. 2021 performance: an R package for assessment, comparison and testing of statistical models. *J. Open Source Softw.* **6**, 3139. (doi:10.21105/joss.03139)

16. Wickham H. 2016 *ggplot2: Elegant Graphics for Data Analysis*. New York: Springer.

17. Lenth RV *et al.* 2025 emmeans: Estimated marginal means, aka least-squares means.

18. Oksanen J *et al.* 2026 vegan: Community ecology package. (doi:10.32614/CRAN.package.vegan)
